# The evolution of the syrinx: An acoustic theory

**DOI:** 10.1371/journal.pbio.2006507

**Published:** 2019-02-07

**Authors:** Tobias Riede, Scott L. Thomson, Ingo R. Titze, Franz Goller

**Affiliations:** 1 Midwestern University, Department of Physiology, Glendale, Arizona, United States of America; 2 University of Utah, National Center for Voice and Speech, Salt Lake City, Utah, United States of America; 3 University of Utah, Department of Biology, Salt Lake City, Utah, United States of America; 4 Brigham Young University, Department of Mechanical Engineering, Provo, Utah, United States of America; 5 Institute for Zoophysiology, University of Münster, Münster, Germany; Emory University, United States of America

## Abstract

The unique avian vocal organ, the syrinx, is located at the caudal end of the trachea. Although a larynx is also present at the opposite end, birds phonate only with the syrinx. Why only birds evolved a novel sound source at this location remains unknown, and hypotheses about its origin are largely untested. Here, we test the hypothesis that the syrinx constitutes a biomechanical advantage for sound production over the larynx with combined theoretical and experimental approaches. We investigated whether the position of a sound source within the respiratory tract affects acoustic features of the vocal output, including fundamental frequency and efficiency of conversion from aerodynamic energy to sound. Theoretical data and measurements in three bird species suggest that sound frequency is influenced by the interaction between sound source and vocal tract. A physical model and a computational simulation also indicate that a sound source in a syringeal position produces sound with greater efficiency. Interestingly, the interactions between sound source and vocal tract differed between species, suggesting that the syringeal sound source is optimized for its position in the respiratory tract. These results provide compelling evidence that strong selective pressures for high vocal efficiency may have been a major driving force in the evolution of the syrinx. The longer trachea of birds compared to other tetrapods made them likely predisposed for the evolution of a syrinx. A long vocal tract downstream from the sound source improves efficiency by facilitating the tuning between fundamental frequency and the first vocal tract resonance.

## Author summary

The larynx is an important valve in the respiratory system of all air-breathing vertebrates that is located at the upper end of the trachea. In some amphibians, in nonavian reptiles, and in mammals, it has also assumed the function of a vocal organ. In contrast, birds have evolved a new and unique vocal organ, the syrinx, which is located at the lower end of the trachea. The selective forces that underlie the evolution of the syrinx as a novel organ have remained unclear. Among all air-breathing vertebrates, birds have the longest necks, and long necks require a long trachea. With a vocal organ at the base of the trachea, this long tube can act as vocal tract resonator and, therefore, can improve the conversion of aerodynamic energy into acoustic energy if fundamental frequency and a resonance frequency are matched. Here, we conducted experiments with simplified physical models, with real birds and a computational simulation in order to investigate the effect of the two different positions of a sound source within the respiratory tract. We find that sound is produced with greater efficiency by a sound source in syrinx position and that favorable interactions between sound source and vocal tract occur with syringeal position. The data provide support for the hypothesis that a selective pressure for high vocal efficiency may have contributed to the evolution of the syrinx in its unique location within the air tract.

## Introduction

Evolutionary novelty in physiological and morphological features can often be traced to specific adaptations that allow organisms to exploit the fitness landscape successfully. The avian clade is characterized by a number of striking synapomorphies, which frequently have been linked to the evolution of active flight [1]. However, many of these avian features did not arise in the context of flight, and the selective regimes that led to their evolution are often poorly understood. The unique avian vocal organ, the syrinx, is such an example [2].

In all air-breathing vertebrates, the larynx regulates airflow and serves as a valve to protect the airways from food and water [3]. In most amphibians, reptiles, and mammals, the larynx has also evolved into a vocal organ [4]. Birds possess a laryngeal valve, but as a sound source, they have evolved a novel structure, the syrinx [5–7]. Even crocodilians, the closest extant relatives of birds, produce sound with a laryngeal source [8,9] and show no modifications at the tracheobronchial juncture, which could be interpreted as precursors of a syrinx [7]. Interestingly, the phonatory mechanisms of the syrinx and larynx are remarkably similar [10], i.e., airflow sets laterally positioned vocal folds into self-sustained vibration [11–15]. The archosaurian shift from producing sound with an organ located at the cranial end of the trachea to a novel structure near the tracheobronchial juncture must have conferred a selective advantage. The nature of the selective forces leading to the formation of a syrinx is still completely unknown.

Is the location of the syrinx at the tracheobronchial juncture linked to the specialized avian respiratory system? The unidirectional airflow through reptilian and avian lungs [16] is in birds associated with the longest tracheas among vertebrates [17,18]. The volume of the avian lung does not change markedly between respiratory phases, as it does in the tidally perfused mammalian lung. Instead, the air sac system functions as bellows and perfuses the parabronchi of the lung with a mostly unidirectional stream of oxygenated air during inspiration and expiration. This flow is thought to arise from two aerodynamic valves at critical conjunctions of the mesobronchi within the lung [19–22]. Although these differences in ventilation of the lung between birds and mammals might indicate that a second air-regulating valve at the tracheobronchial juncture in the interclavicular air sac is critical for regulating airflow, there are no experimental data to substantiate this hypothesis. Birds with experimentally deactivated syringeal muscles can breathe without difficulty in a lab setting, but of course, some more metabolically demanding dynamic behaviors such as flight have not been tested directly. Irrespective of whether an air-regulating valve evolved prior to the vocal function, the switch of vocal organ from a laryngeal to the syringeal position needs an explanation.

Selective pressures related to vocal production must therefore have been in play to cause this switch in vocal organ. In this study, we ask whether the syringeal sound source provides greater vocal efficiency than a larynx because the location of the sound source leads to differences in how self-sustained vocal fold vibrations interact with vocal tract resonances. Specifically, we predict that a sound source in syringeal position converts aerodynamic energy into acoustic energy more effectively because of more favorable interactions with the upper vocal tract. We tested this hypothesis through a series of experiments, which were designed to inform our understanding of the major difference between larynx and syrinx—their location within the respiratory system. Two modeling efforts were used to test whether positioning a sound source upstream (as in the syrinx) or downstream (as in the larynx) of an elongated tube (the trachea) has a significant effect on the efficiency of sound production. The modeling approaches cannot be replicated one-to-one in vivo because a syrinx or larynx, respectively, cannot be moved freely up and down the tracheal tube. However, a complementary experimental approach is the manipulation of tracheal length to investigate the effect of tracheal resonances on the syringeal sound source. The influence of acoustic airway pressures on vocal fold vibration in the syrinx was therefore studied in bird cadavers by changing the tracheal length above the sound source by tube extensions. The in situ experiments helped to investigate the role of an interaction between sound source and length of the vocal tract filter.

In the following section, we lay theoretical groundwork for exploring how the position of a sound source within the respiratory tract determines vocal output characteristics.

### Acoustic theory of self-oscillating valves in tubes and ducts

Direct visualization of the sound-producing larynx [23] and syrinx [12] and experiments with an excised larynx [24–27] and syrinx [11,13,15] confirm that both organs function as self-oscillating valves driven by airflow. The primary sound then travels along the respiratory tract above the source, which for laryngeal phonation consists of oral, nasal, and pharyngeal cavities [28–30] and in birds of the tracheal tube, larynx, oropharyngeal–esophageal cavity, and beak [31–34]. An obvious, major difference between the laryngeal and syringeal design is the relative length of the airway above and below the sound source. This difference has important consequences for how the vibrating tissue of the sound source and the air column in the vocal tract interact. The air columns above and below each sound source can affect the way energy is conveyed from the aerodynamic airflow to the vibrating tissue masses.

Titze [35] used a surface-wave model to show how energy from the airstream becomes coupled to the vocal folds. The driving force for tissue vibrations in the syrinx and larynx is lung pressure. The lowest driving pressure required for triggering tissue vibration is referred to as phonation threshold pressure (*p*_*th*_) and provides an important estimate of the energy conversion. Phonation threshold pressure was derived as

$$p_{th}=k_{t}\left(\frac{\rho }{2}\right){\left(\frac{B}{2Ll_{t}}\right)}^{2},$$ (1)

in which *k*_*t*_ is a dimensionless pressure coefficient (average of about 1.1 over a vibration cycle), *ρ* is the air density, *B* is vocal fold tissue damping, *L* is vocal fold length, and *l*_*t*_ is the acoustic inertance of the downstream tube. Inertance is the sluggishness (or inertia) of the vocal tract air column. As the supraglottal column of air is driven forward and backward by airflow emerging from the glottis, the sluggishness of the air column creates an acoustic pressure that helps the vocal folds in their self-sustained oscillations [28, Chapter 4]. The phonation threshold pressure thus varies inversely with the square of inertance. Greater inertance lowers the phonation threshold pressure, making it easier to produce vocal fold oscillations. For a tube that is acoustically short (i.e., much less than a quarter of a wavelength), the inertance can be expressed simply as

$$l_{t}=\frac{\rho L_{t}}{A_{t}},$$ (2)

in which *L*_*t*_ is the length of the tube and *A*_*t*_ is the cross-sectional area. Eq 2 shows that a longer and narrower tube produces greater inertance. When the tube is lengthened much beyond a quarter wavelength, however, standing waves can be produced due to reflections from the distal end of the tube, and the inertance then becomes frequency dependent. In fact, for some frequencies, the vocal tract may become compliant rather than inertive, in which case supraglottal pressures hinder self-sustained oscillations of the vocal folds. To maximize energy transfer, it is then important that the bird develops an appropriate length–frequency combination that produces inertance at the input of the trachea, which passerine songbirds indeed do by matching fundamental frequency and first formant frequency [32]. Tissue oscillations are maintained much more easily, i.e., with lower subglottal/subsyringeal pressure, at frequencies for which the air column is inertive rather than compliant, which generally occurs at frequencies below a resonance frequency of the tube. Tracheal lengths that are slightly below one-fourth, three-fourths, one and one-fourth, one and three-fourths, etc. wavelengths are theoretically ideal for a tube closed at one end and open on the other end. The exact boundary conditions may differ, however, with variable glottal impedance and radiation impedance.

The effect of the vocal tract on self-sustained oscillation is reversed for a subglottal/subsyringeal airway system. Fletcher [36] expanded the theory of self-oscillating valves in a tube by including valves with both lateral and longitudinal degrees of freedom (relative to the airflow and an upstream acoustic tube). For the important lateral degree of freedom in vocal fold vibration, inertance below the larynx was not favorable to vocal fold vibration, raising the threshold pressure rather than lowering it. Titze [37] showed that combining a compliant system below (i.e., upstream) the sound source with an inertive system above (i.e., downstream) the sound source provides the best assistance to self-sustained oscillation. A second benefit obtained from an inertive tube is the delay of the peak acoustic airflow through the glottis relative to the peak excursion of the lateral vocal fold movement, generating a “skewing of the airflow waveform” toward a sawtooth shape. In human subjects, these interactions between source and filter have different effects on the voice during spontaneous vocalization, including an increase in overall intensity, an increase of the higher harmonic energy, or an increase in the probability of nonlinear phenomena [38].

In summary, theoretical acoustic analysis predicts that (1) subglottal/subsyringeal inertance raises the phonation threshold pressure but increases glottal waveform skewing, the benefit and costs of which can offset each other, and (2) that supraglottal/suprasyringeal inertance lowers oscillation threshold pressure and increases glottal waveform skewing, an additive beneficial effect. Thus, a sound source deeper in the airway would appear to have an advantage, assuming that the extra energy losses in the longer transmission system do not negate the additional energy converted.

## Materials and methods

We conducted three tests to investigate whether the position of the sound source within the respiratory tract affects the primary sound production.

### Ethics statement

All procedures using birds were approved by the Institutional Animal Care and Use Committee of the University of Utah (protocol number 16–03014). The protocols are in compliance with the Animal Welfare Act regulations and Public Health Service Policy. The university maintains accreditation by the Association for the Assessment and Accreditation of Laboratory Animal Care International.

### Physical model

In the first study, we used a model that consists of two vocal folds constructed from silicone [39]. The silicone model was a single-layer representation of the vocal folds or of labia and membranes in a syrinx [40,41]. The model cross section was uniform in the dorsoventral direction up to the point of intersection with the tube wall. Resonance properties of the model were determined by placing the model on a small shaker, which created small amplitude vibrations from 5 to 500 Hz. The beam of a laser Doppler vibrometer (Polytec Scanning Vibrometer; Polytec, Inc.) was positioned on one vocal fold in order to measure its frequency response. A Fourier analysis of the model vibration yielded a fundamental frequency of 78 Hz.

A simple physical model was used that neither includes detailed features of syringeal or laryngeal morphology nor imitates the respective respiratory anatomies. By not including features such as air sacs and accessory elastic tissue components or musculature, we limit the number of variables to sound source position and trachea length. This provides a clear and simple test of whether one location of a sound source confers an acoustic advantage over another. Physical models such as that used here have been tested in previous studies investigating different questions related to human voice production and biomechanics [26,42–44].

**Fig 1:**
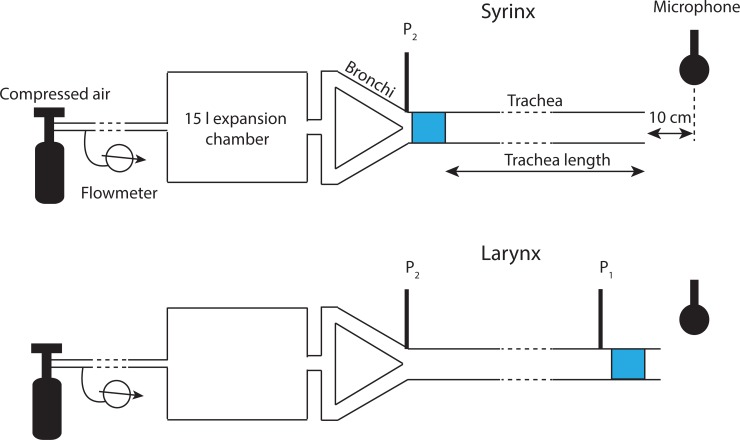
Schematic (not to scale) of the experimental setup testing a physical model (blue region) in a syrinx or larynx position. The microphone was placed 10 cm downstream from the opening of the vocal tract. Pressure transducers (P1, P2) were placed below the respective sound sources. A 15l expansion chamber simulated acoustic properties of the lung. Flow rate was measured upstream from the expansion chamber. Trachea length was varied between 0 cm and 248 cm.

The two vocal folds were mounted inside a 1-inch inner-diameter PVC ring that could be coupled to a 1-inch inner-diameter PVC tube. Blowing compressed air through a tube containing the vocal folds initiated vocal fold vibration and created sound. The sound source (i.e., the ring containing the vocal folds) was placed either at the upper (larynx) or lower (syrinx) end of a PVC tube (Fig 1). The PVC tube length was varied from 0 to 248 cm in a stepwise fashion. For the syringeal position, the sound source was placed 2 cm above a y-shaped tube simulating the bronchial bifurcation. The laryngeal sound source was placed at the downstream end of the trachea and was equipped with a short 15-cm-long vocal tract above the source.

Air was supplied from a tank with compressed air through a 5-m Silastic tube, which was connected to both bronchial tubes. Average airflow was measured 3 m upstream from the tracheal bifurcation in the Silastic tubing with a rotameter mounted in series (KING instruments company, Garden Grove, CA; maximum flow rate: 4 SCFM) and in one bronchial tube (flow meter MC-5SLPM-D-15PSIA; Alicat Scientific).

Pressure was measured below each sound source (Fig 1) and, in the case of the laryngeal sound source, at the lower end of the trachea. The latter allowed us to monitor the pressure gradient along the tracheal tube, which never exceeded 20 Pa/m. Calibrated pressure measurements were made through small (1-mm inner diameter) stainless steel tubes mounted into the wall of the PVC tubing and connected through Silastic tubing to a pressure transducer (model FHM-02PGR-02; Fujikura, Tokyo, Japan).

A calibrated microphone (GRAS Sound and Vibration, Denmark; pressure microphone 40 AG, preamplifier 26 AK and 12 AD power module) was placed perpendicular to the vocal tract opening at a distance of 10 cm. Sound, airflow, and pressure signals were recorded through a multichannel AD-acquisition board (NI DAQ). Signals were digitized at 44.1 kHz sampling rate with Avisoft Recorder software (Avisoft, Berlin, Germany).

We measured driving pressure, tracheal airflow, sound pressure level, and fundamental frequency and estimated vocal efficiency as the ratio of radiated acoustic power (*P*_*r*_) over aerodynamic power (*P*_*a*_):

$$E=\frac{P_{r}}{P_{a}}.$$ (3)

Radiated power is a measure of the amount of aerodynamic energy converted into acoustic energy and radiated into the air per second (in Watts). Assuming spherical radiation, it can be calculated from the sound pressure level (in dB) at a radius *R* from the opening of the vocal tract (lips or beak in animals; open tube end in our experimental setup):

$$P_{r}=4\pi R^{2}\times 10^{(SPL-120)/10},$$ (4)

in which *P*_*r*_ is the radiated power, *R* is measured as distance between microphone and the opening of the vocal tract tube (here, 10 cm), and *SPL* is the sound pressure level (dB). Aerodynamic power is derived as the product of mean flow rate and subglottal/subsyringeal pressure:

$$P_{a}=p_{s}V,$$ (5)

in which *P*_*a*_ is the aerodynamic power (Watts, W), *p*_*s*_ is the pressure below the sound source (Pascal, Pa), and *V* is the mean flow rate (cubic meters per second, m^3^/s).

The vocal fold model, either in syringeal or laryngeal position, was coupled with 18 different tracheal lengths. The segment lengths were chosen so that the first tracheal resonance is either lower, higher, or equal to the eigenfrequency of the physical model of 78 Hz. The first and second resonances are provided in S1 Table.

Studies linking fundamental frequency and body size [45] or body size and tracheal length [18] suggest that our vocal fold model would be that of a 2 to 30 kg bird with a tracheal length of approximately 40 cm. Therefore, the simulations with very short and very long tracheal lengths are representative for birds with extreme trachea morphologies [46]. It is the range around 40-cm trachea length that resembles most realistically a bird-like situation with average tracheal length.

### Computational simulation

In a second study, we investigated the interdependency between sound source position, glottal efficiency, and vocal tract length by computational simulation. In agreement with the physical model construct, we did not make the self-oscillating sound source specific to any species or gender nor did we include any layered tissue morphology. Rather, we used a simple generic self-oscillating tissue surface model. The “vocal folds” were defined by five serially coupled sections of a soft-wall tube (1.6 mm each section in the caudal–cranial direction), giving the vibrating tissue an overall thickness of 8.0 mm. The sections had elliptical cross sections. The minor diameters (also known as the prephonatory glottal widths) were 1.0, 0.8, 0.6, 0.6, and 1.0 mm, caudal to cranial, whereas the major diameters (also known as vocal fold length, ventral to dorsal) were all 10 mm. The viscoelastic properties of the wall were patterned after the two-section model of [47], with a Young’s modulus = 4.0 kPa, a shear modulus = 1.0 kPa, mass per unit area = 0.3 g/cm^2^, and a damping ratio of 0.1. This produced a natural tissue frequency of 130 Hz in each section.

Fluid flow and acoustic wave propagation in all airway sections (including the source sections) were calculated on the basis of conservation of momentum and mass transfer using the Navier–Stokes and continuity equations for nonsteady, compressible airflow [48,49]. Fluid pressures on the surfaces of the five source sections provided the driving forces for self-sustained oscillation. The tracheal length was varied from 22 cm to 154 cm in steps of 22 cm. In case of the syrinx position, an additional 1.0-cm section length was added between the source and the bronchial termination, while for the larynx position, an additional 1.0-cm length was added between the source and the mouth radiation. Radiation from the mouth was computed with the piston-in-a-spherical-baffle model [49]. The cross-sectional area of the tube was length dependent, as defined below. The viscoelastic wall properties for all sections except the vocal fold sections were chosen according to Titze and colleagues [49]: Young’s modulus = 9.62 kPa, shear modulus = 1.67 kPa, mass per unit area = 1.5 g/cm^2^, and damping ratio = 1.26.

The power calculations were as follows:

$$P=\frac{1}{N}\sum_{n=1}^{N}\left(p_{n}U_{n}\right)\;\mathrm{total}\;\mathrm{power},$$ (6)

$$P_{DC}=p_{DC}U_{DC}\;\mathrm{steady}\;\mathrm{flow}\;(\mathrm{DC})\;\mathrm{power},$$ (7)

$$P_{AC}=P-P_{DC}\;\mathrm{acoustic}\;(\mathrm{AC})\;\mathrm{power},$$ (8)

in which *n* is the time sample index, *N* is the number of samples simulated (22,050 in a 0.5-s window), *p*_*n*_ is the instantaneous pressure in a given section, *U*_*n*_ is the instantaneous flow rate, *p*_*DC*_ is the steady (DC) pressure, and *U*_*DC*_ is the steady (DC) flow rate, computed as time averages over the 0.5-s window. The instantaneous powers *p*_*n*_*U*_*n*_ in Eq 6 varied dramatically in the vocal fold sections where self-oscillation took place. Therefore, to get a representation of the mean values of intraglottal pressure, flow, and power, the calculations in Eq 6 were averaged over the five adjacent vocal fold sections.

The efficiency was computed as the acoustic power delivered to the mouth divided by the total input power. This was different from the efficiency calculation with Eq 3 for the physical model. Mouth power gave a more accurate difference calculation in Eq 8. Radiated power was many orders of magnitude lower than the input power when the tube was more than 1.0 m long. Thus, the magnitudes of the efficiency calculations between the physical model and the computational model are not comparable, but the variations with length and source position are comparable.

Unlike in the first experiment with the physical model, tracheal diameter was adjusted with changing tracheal length. This simulates conditions in avian archosaurs more realistically. As indicated in Eq 2, not only the length but also the diameter of the downstream airway affects tissue vibration characteristics. The relationship between tracheal length (*TL*) and tracheal diameter (*TD*) was estimated with Eq 9 following published empirical data [18]:

TD=(0.24*TL)+0.1. (9)

We used tracheal diameter to estimate vocal fold length. We assume that vocal fold length is approximately equal to tracheal diameter [45]. According to Eq 9, a 1-cm tracheal diameter would suggest a tracheal length of 41 cm in a hypothetical bird, i.e., similar to the physical model. Tracheal lengths much shorter or longer could be representative of extreme tracheal morphologies.

### In situ syringeal sound production with variable tracheal lengths

In a third study, we investigated the effect of tracheal length on sound production by a syrinx in situ. The approach of the in situ syrinx has been proven effective [11,50–53]. It is important to perform these experiments in situ because excised syringeal preparations may reveal unnatural vibratory behavior of the labia [54].

If the length of the airway above a sound source is a critical factor determining the acoustic output of a bird, we expect systematic changes associated with tracheal length changes. The syrinx can be phonated by blowing air into the posterior thoracic or abdominal air sac. The experiments were performed in freshly killed birds. Five male specimens from each of three species (chicken, *Gallus gallus*; budgerigar, *Melopsittacus undulatus*; and zebra finch, *Taeniopygia guttata*) were used. Birds were euthanized with an overdose of Ketamine/Xylazine. Compressed humidified and warm air was injected into the right posterior thoracic or caudal air sac through a Silastic tube. A microphone was placed 10 cm downstream from the cranial opening of the trachea.

We measured fundamental frequency while phonating the syrinx. Subsyringeal air sac pressure, which is proxy for the driving pressure of the sound source, was measured in the right anterior thoracic air sac by inserting a flexible cannula through a small hole in the body wall (Silastic tubing; 1.65 mm o.d., 6 cm length). The free end of the tube was connected to a piezoresistive pressure transducer (model FHM-02PGR-02; Fujikura, Tokyo, Japan).

The numerical data used in all figures are included in S1 Data.

## Results

### Physical model

**Fig 2:**
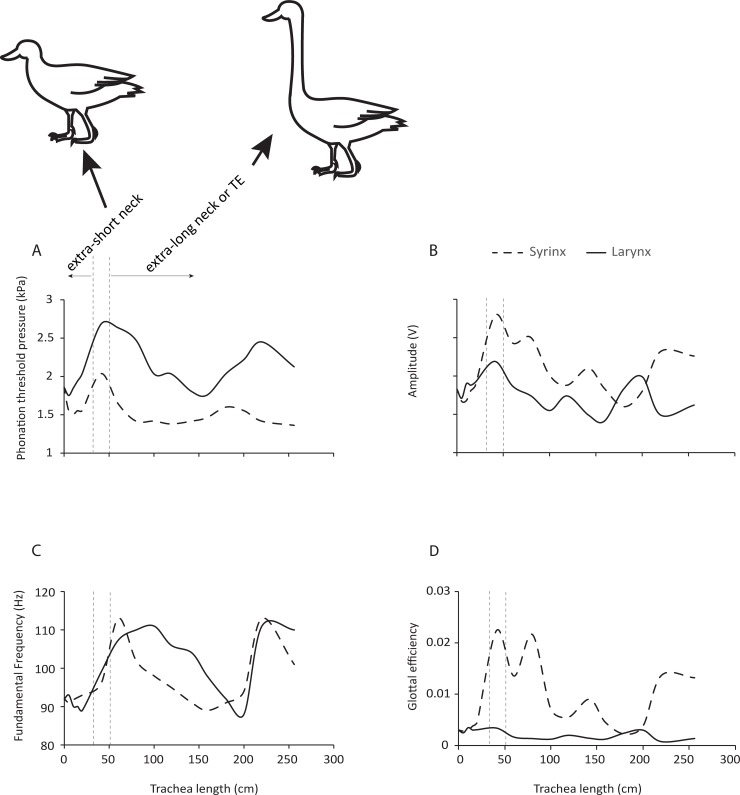
Sound production is more efficient when the sound source is in a syringeal position. (A) PTP was consistently lower in the syrinx than in the larynx. (B) The syringeal sound source generated higher sound intensities (depicted as Amplitude, relative change in output voltage of microphone signal) than the larynx for most tracheal lengths. (C) The fluctuating fundamental frequency is evidence for the strong interaction between sound source and vocal tract filter. (D) Glottal efficiency of a laryngeal or syringeal sound source at different tracheal lengths. Between 40 and 80 cm tracheal length, the effect is most dramatic. The two vertical dashed lines indicate a tracheal length range (40 cm ± 10 cm), which is most likely to correspond with a sound source of 1 cm vocal fold length used in this study. This tracheal length corresponds to an avian archosaur of approximately 20 to 30 kg body mass [18]. Tracheal lengths much shorter or much longer than this range could correspond to a few extreme exceptions in which the trachea is very short or very long. Numerical data used in this figure is included in S1 Data. PTP, phonation threshold pressure; TE, tracheal elongation.

Phonation threshold pressure varied between 1.4 and 2 kPa for the syringeal and between 1.7 and 2.7 kPa for the laryngeal position of the physical model source. Phonation threshold pressure was lower for the syrinx for all tracheal lengths tested (Fig 2A). Very high pressures were required to trigger phonation in the larynx for tracheal lengths between 36 and 86 cm (Fig 2A) when the vocal fold eigenfrequency was located to the left of the first tracheal resonance.

For a very short trachea and for a tracheal length around 2 m, the laryngeal and syringeal sound sources generated comparable sound intensities, but in all other conditions, the syringeal source emitted louder sound (Fig 2B).

Tracheal resonances influenced the vibration rate of both sound sources (Fig 2C). Increasing tracheal length was first accompanied by increased fundamental frequency, which peaked at approximately 60 cm tracheal tube length and then decreased with longer tracheae.

Glottal efficiency of the syrinx was greater across almost all tracheal lengths (Fig 2D). The difference was most dramatic in the range between 40 and 80 cm. Efficiency of the laryngeal sound source was comparatively flat across all tracheal lengths.

In sum, the findings support the hypothesis that the position of the sound source within the respiratory tract critically affects vocal parameters. For a given body size (approximated at 20 to 30 kg body mass associated with a tracheal length of 40 cm), the syringeal position is more efficient in energy conversion.

### Computational simulation

**Fig 3:**
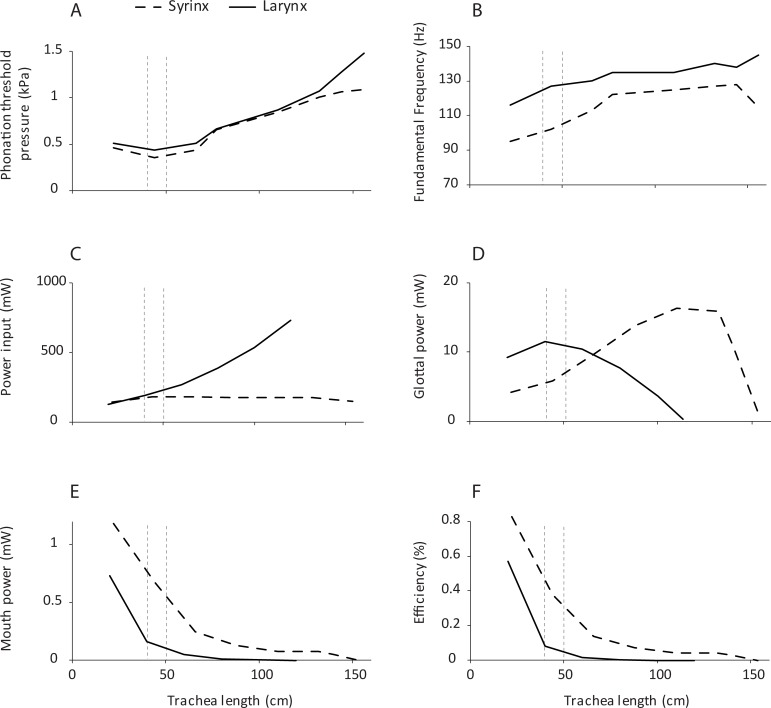
The effect of trachea length as studied by computational simulation. Differences between the syrinx and larynx position in phonation are shown for threshold pressure (A), fundamental frequency (B), power input into trachea (C), glottal power (D), power at the mouth (E), and efficiency (F). Efficiency was computed as the mouth power divided by the input power. The two vertical dashed lines indicate the tracheal length range (40 cm ± 10 cm), which is most likely to correspond with a sound source of 1 cm vocal fold length used in this study (see Fig 2). Numerical data used in this figure is included in S1 Data.

Phonation threshold pressure varied between 0.4 kPa and 1.0 kPa for the syringeal position and between 0.5 and 1.5 kPa for the laryngeal position. This pressure was lower for the syrinx for all tracheal lengths tested (Fig 3A). Fundamental frequency was categorically lower for the syringeal position. This is in agreement with analytical predictions that greater supraglottal acoustic inertance lowers F0. Glottal efficiency was categorical greater for the syringeal position than for the laryngeal position. We also tested to what degree source position affects vocal parameters with the computational model. The critical length for the 130-Hz natural frequency of oscillation of the computational model was 67.5 cm, for which the tube resonated at a quarter wavelength at 130 Hz. For all lengths below this critical length, the vocal tract acoustic reactance is inertive, which means that there is a favorable source–vocal tract interaction [37]. This favorable interaction explains why in Fig 3, in the region below 67.5 cm, the phonation threshold pressure is lower and fundamental frequency is slightly lower. Vocal tract inertance adds effective mass to the coupled oscillator system and therefore lowers fundamental frequency. Furthermore, acoustic power at the mouth is higher, and vocal efficiency is higher than in the region above the critical length. Phonation threshold pressure was on the order of 0.5 kPa in the inertive region and rose to 1.2 kPa in the noninertive region for both the syrinx and the larynx position. It reached its minimum at a tracheal length of about 40 cm (Fig 3A).

All variations with tube length and source position in Fig 3 are not as large as in the physical model. This is attributable to four important differences: (1) the wall properties of the tube, (2) a more realistic simulation of tracheal diameter, (3) the difference in vocal fold geometry and material properties, and (4) the difference in radiation from the tube. The computational model used soft walls throughout, while the physical model used a hard-wall PVC tube. The acoustic energy levels in hard-wall tubes are much greater than in soft-wall tubes, increasing the degree of interaction between the source and the vocal tract. The interaction in the computational model was also lessened by the fact that the cross-sectional area of the tube increased with length. It is well-known that vocal tract pressures are scaled by the characteristic tube impedance *ρc/A*, where *ρ* is the air density, *c* is the speed of sound, and *A* is the cross-sectional area. Greater cross-sectional area lowers all vocal tract pressures. Furthermore, the way vocal folds respond to airflow is governed by factors such as geometry, layer structure, and viscoelastic properties. While the physical and computational vocal fold models shared similar characteristics, the effects of geometric and material property differences are not fully understood. Finally, the radiation from a tube without a baffle, which was the case in the physical model experiments, may differ from radiation from a piston in a spherical baffle, which was assumed in the computation.

### In situ syringeal sound production with variable tracheal lengths

**Fig 4:**
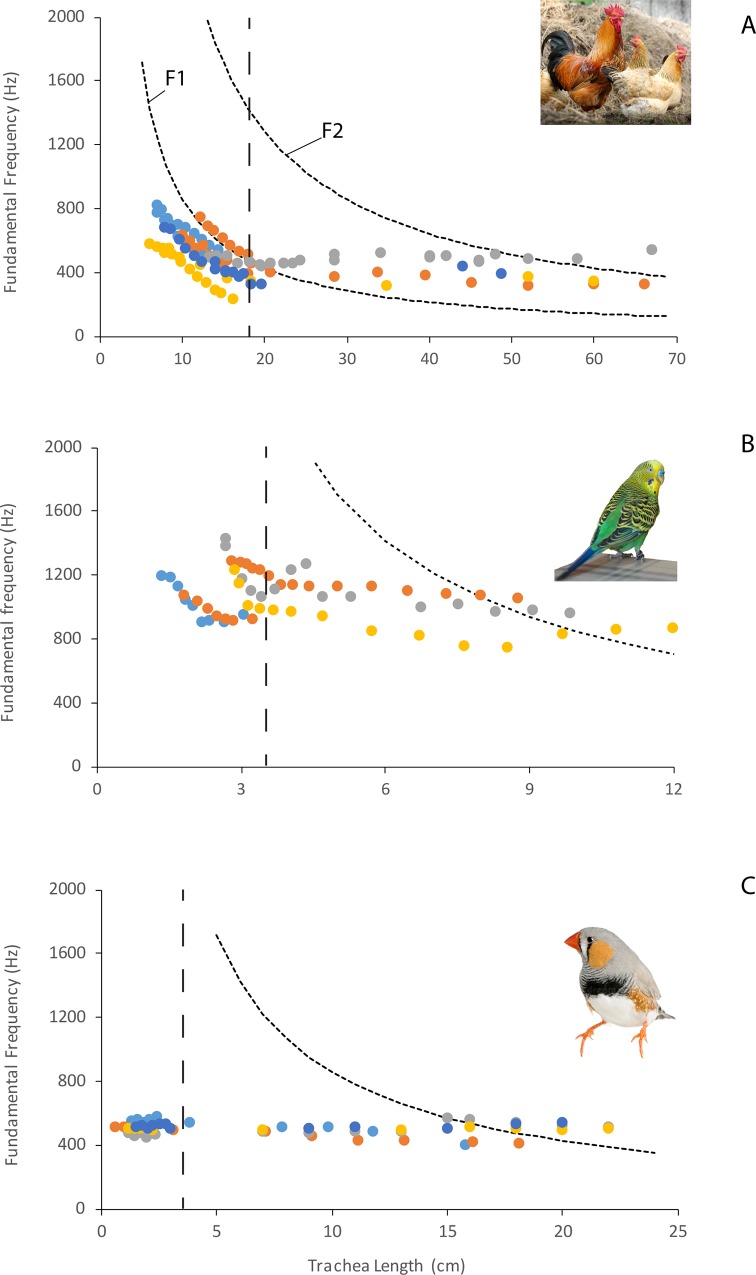
(A–C) Trachea length was stepwise elongated (to the right of dashed line) or shortened (to the left of dashed line). In chicken and budgerigars, fundamental frequency increases in response to trachea shortening and slightly decreases in response to trachea elongation. In the zebra finch, very small changes were observed in fundamental frequency. However, vocal quality changed sometimes dramatically (e.g., occurrence of nonlinear phenomena). The decrease of fundamental frequency in the inertive reactance region below F1 (primarily in the chicken) is in agreement with theory. Inertance of the vocal tract effectively adds mass to the coupled oscillating system, thereby lowering F0. The closer F0 is to F1, the greater the inertance and hence the greater the F0 drop. Why did the zebra finch apparently exhibit only type 1 coupling throughout shortening and lengthening? There are morphological differences between the structures of the vocal folds of the three bird species that could explain this. The initial length of the trachea is shown by the vertical dashed line. Numerical data used in this figure is included in S1 Data. F1, first formant

A total of four chickens, five budgerigars, and five zebra finches were successfully phonated. The stepwise shortening of the trachea was accompanied by an increased fundamental frequency in chickens and budgerigars (Fig 4A and 4B). In zebra finches, fundamental frequency remained relatively constant (Fig 4C). The elongation of the trachea with tubes that fitted the respective trachea was associated with a decrease of fundamental frequency in chickens but not in budgerigars and zebra finches (Fig 4A–4C). In male zebra finches, we observed nonlinear phenomena in the phonations more frequently if tracheal length resonance was near the fundamental frequency.

## Discussion

The results presented here provide two findings that enhance our understanding of which selective pressures might have led to the evolution of the unique avian syrinx. First, the physical and the computational models of the sound source show that sound production with a source in the syringeal position can be dramatically more efficient than in the laryngeal position. Second, sound production by the in situ syrinx is affected by vocal tract resonance. In the following, we will discuss how these results might be important for shedding light on the evolution of the syrinx.

In our physical model, the use of an identical sound source in both positions provides strong support that the additional length of the vocal tract above the sound source plays a significant role in improving vocal efficiency. Importantly, these differences between larynx and syrinx emerged in both modeling approaches and without the need for incorporating other avian specializations of the respiratory system that likely improve vocal efficiency. Most notably, the syrinx resides in the interclavicular air sac, whose pressure conditions may affect sound production by pushing the vibrating tissue into the airstream and thus likely lower the phonation threshold pressure with increased adduction.

Although the influence of airway characteristics upstream and downstream from a sound source on the sound generation mechanism has been discussed before [37,55,56] and has been most clearly documented for the human voice [38,57–60], unequivocal experimental evidence for source–tract interactions has not been presented for birds. Our data from three species show that these interactions do play a role, but their prominence may differ substantially between species. The differences are either based on different types of interaction between sound source and vocal tract or on different strengths of one type of interaction. In two species, chicken and budgerigar, fundamental frequency was tightly linked to the first tracheal resonance (F1 in Fig 4). As the first tracheal resonance increased and moved away from the natural source frequency (trachea shortened), the fundamental frequency of self-sustained oscillation increased too, i.e., it was less bent downward by the inertive acoustic load of the first resonance. In the third species, the zebra finch, no such change was observed. Instead, there was an increased occurrence of nonlinear phenomena (e.g., subharmonics and frequency jumps) when the trachea was shortened.

This interpretation is further supported by experiments with a variety of approaches, which were undertaken to determine the mechanism of sound production in birds. In various studies using different types of physical models, the vibration frequency [61–64] showed strong coupling to the first resonance of the downstream vocal tract. In situ phonation experiments in bird cadavers further support our finding that species differ in the strength and/or nature of the coupling. Whereas fundamental frequency changed around resonance frequencies of the trachea in several species—*Gallus gallus domesticus* [50,11,65], *Grus grus* [11], *Meleagris gallopavo* [50], *Anser anser* [52]—it did not to the same extent in one Great-horned owl (*Bubo virginianus*) [51]. Furthermore, spontaneously singing passerine songbirds tested in a heliox atmosphere did not show marked changes in frequency [66], suggesting that, by altering resonance, vibration frequency is not markedly affected.

Our data and published evidence [63] in the budgerigar show an interesting additional detail. In intact birds singing in heliox, fundamental frequency changed very little. However, once the syringeal muscles were denervated, fundamental frequency increased dramatically and followed the first tracheal resonance, suggesting that the coupling of source and tract is under neural control. In our phonation experiments on this species, we also see effects of strong coupling on sound frequency in the absence of neural control.

**Fig 5:**
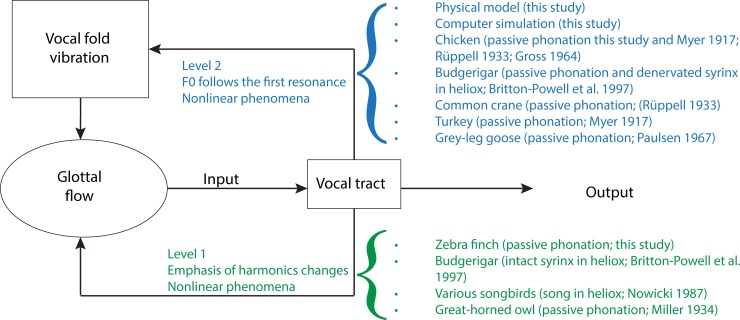
The interaction between sound source and filter is conceptualized as either Level 1 or Level 2 interaction. Level 1 interactions are characterized by effects on the glottal flow, which will lead to acoustic effects, including changes in harmonic emphasis and/or the occurrence of nonlinear phenomena. Level 2 interactions are characterized by effects on vocal fold vibrations, which will lead to changes in fundamental frequency and/or also the occurrence of nonlinear phenomena.

In principle, tracheal length, i.e., the size of the air column above the sound source, can have an effect on the vibrating structures in two ways [37,67,68,69]. “Level 1” interaction affects the glottal airflow but has no effect on vocal fold movement, whereas a “Level 2” interaction affects both airflow and vocal fold movement (Fig 5). Whenever fundamental frequency and amplitude of vibration change with vocal tract adjustments, Level 2 interaction is indicated. If only the sound intensity and spectral content change without accompanying changes in vocal fold movement, Level 1 interaction is indicated. The output of a system with Level 1 interaction shows changes in the amplitude of various harmonics. The frequency output of a system with Level 2 interaction is characterized by first bending downward as it approaches the resonance frequency from below. It then locks onto the resonance frequency and follows it if the coupling is strong. With very strong interaction, occurrence of nonlinear phenomena (bifurcations) in the acoustic output can make predictions of the frequency change more difficult [59]. Nonlinear phenomena can be evident in the glottal flow signal (Level 1 interaction, Fig 5) as well as in the tissue movements of vocal folds (Level 2 interaction, Fig 5). The differentiation of the two types of interaction cannot be made accurately based on the acoustic output alone but requires additional evidence, i.e., direct visualization of the vocal folds [68], preferably in vivo.

Our results in chicken and budgerigar suggest Level 2 interaction because fundamental frequency follows the first resonance as the trachea becomes shorter. In other species that have been investigated, the interpretation is less clear because features of both types of interaction were found in the acoustic output (Fig 5). A possible explanation for these different source–tract interactions of different species may lie in morphological differences in the structure of the vocal folds. Vocal folds in birds vary substantially in design, ranging from thin membranes (e.g., in many Galloanseriformes and parrots [46]) to thick multilayered structures in Passeriformes [40,41]. The layer structure in passeriform vocal folds is a good predictor of a species’ fundamental frequency range used during singing [20].

### The evolution of the syrinx: A selective pressure for increased efficiency?

Our approach has addressed the origin of the syrinx as opposed to its diversification. It is therefore imperative to assume a simple sound source, rather than the diverse morphologies found in extant birds. Once the relocation of the sound source had occurred in an as of yet unknown ancestor of Aves, the further diversification may have explored many different avenues for further increasing vocal output, such as two sound sources, different interactions with the upper vocal tract, etc. The different mechanisms of interaction suggest a possible, albeit speculative, scenario for the origin of the syringeal sound source within Aves. If strong interaction leads to less control or uncertainty in fundamental frequency because of a sparsity of vibration modes, then perhaps the original syrinx represented a vocal organ with Level 2 interaction. Typically, Level 1 interaction arises in conjunction with histologically more complex vibratory tissue [37]. In extant birds, more complex vocal folds evolved, i.e., in songbirds [20], while other groups, i.e., parrots or Galliformes, possess thin membranes but display variable degrees of muscular control of the syrinx [46]. The heliox data in intact and denervated budgerigars suggest that muscular control of the syrinx can modulate source–tract coupling.

This initial investigation of possible selective advantages of a syringeal location of the sound source also highlights that the evolutionary origin of novelty can be addressed with specific tests of hypotheses about selective scenarios. Our data show that one likely selective advantage of the syringeal position is increased efficiency. The ability to generate loud sounds is important for long-range acoustic communication and in the context of courtship and territory defense [70, 71]. Thus, both natural and sexual selective forces may have contributed to the evolution of the avian syrinx. To what degree an early syrinx may have coexisted with a laryngeal sound source remains to be determined.

The modeling and the experiments conducted here deliberately constitute a test of a limited and small set of parameters rather than a physical replica of the avian vocal organ, with all its complexity. While this minimalist approach is likely to inform about a possible selective advantage for the switch in source location, it does not include a thorough test of other selective scenarios and does not explore other likely adaptations for increased efficiency in extant birds. Therefore, future work will have to test whether the dramatic efficiency advantage of a syringeal position is maintained for various syrinx designs or if other variables emerge as the main targets of selection. Syrinx morphology shows remarkable diversity, including features such as multiple sound sources [53], multilayered vocal fold design [41], or changes in vocal tract design and motility [32]. All of these features affect efficiency, and we do not know how they are influenced by trade-offs between vocal efficiency and those other acoustic features. Nevertheless, our approach presents a first test and sets the stage for testing additional hypotheses related to syrinx origin and syrinx diversification.

### Transition from larynx to syrinx

The current study highlights vocal efficiency as an important selective force that may have played a role in the evolution of the syrinx as a vocal organ. The timing of the transition from larynx to syrinx in the theropod lineage leading to modern birds is unknown prior to 66–68 million years ago [7]. Whereas clarification awaits new fossil data, the results from this study allow some speculation about a possible scenario and therefore the timing of syrinx evolution.

The response curves in both the physical model and computational simulation (Fig 2 and Fig 3) demonstrate that the interactions between sound source and vocal tract are complex and nonlinear. There are regions that represent local optima and others that are unfavorable for sound production. For example, phonation threshold pressure is lowest in a region of tracheal length 75–150 cm (Fig 2A) and 40–70 cm (Fig 3A). This demonstrates that for a given sound source, a certain tracheal length range is optimal, and both achieved maxima that are higher for the syringeal than the laryngeal position.

There are also regions in which the rate of change reaches a maximum. For example, the region between 50 and 100 cm trachea length appears to be such an inflection point. Glottal efficiency begins to increase at about 100 cm and with smaller trachea lengths. Again, this is more dramatic for the syrinx than for the larynx (Fig 2D and Fig 3F). Our data therefore highlight the possibility that the evolution of a simple syrinx may be tied to a specific constellation of body size and vocal fold morphology. The theropod lineage leading to birds underwent sustained miniaturization of body size and rapid diversification [72], and in these processes, it is possible that combinations of body size–dependent vocal tract length and sound frequencies favored the evolution of a novel vocal organ. For the sound source used in our study, a tracheal length between 50 and 100 cm yields higher vocal efficiency for the syrinx than the larynx.

An important question for the evolution of the syrinx is to what degree the advantageous interaction between sound source and vocal tract resonance, shown here for a specific size, can be generalized to other body sizes? We postulate that this favorable interaction is not limited to a specific size; as long as the tracheal resonance remains above but close to the fundamental frequency, a favorable suprasyringeal inertive compliance will provide the best assistance to self-sustained oscillation [37]. While the one available study on avian tracheal length suggests that also in small birds, the trachea remains relatively longer than in similar sized other vertebrates [18], a broader and more systematic sampling of avian tracheal anatomy seems warranted.

However, even if the exceptional avian body size–trachea length relationship did not hold for smaller birds, many modern birds possess intrinsic syringeal musculature and are able to modulate vocal fold tension, i.e., fundamental frequency can be actively adjusted to remain close and below the tracheal resonance. Furthermore, small birds have three potential mechanisms for dynamically adjusting their vocal tract resonances: (1) tracheal length changes [73], (2) size changes of the laryngeal aperture [31], and (3) size changes of the oropharyngeal–esophageal cavity [32]. The long tracheal vocal tract in addition to dynamic filter components might allow birds of all sizes to easily maintain tuning of the vocal tract resonance to a quarter wavelength of the fundamental frequency.

### Implication for vertebrate vocal communication or why did only birds evolve a syrinx?

If the syrinx is so much more efficient, why do other groups such as nonavian reptiles, frogs, and mammals continue to use the larynx as sound source? The avian trachea is acoustically longer than those of mammals, nonavian reptiles, and frogs. “Acoustically long” means that tube length is near the quarter wavelength of the fundamental frequency of the sound source (see Introduction, Eq 2). The first ancestral bird with a syrinx most likely produced a low fundamental frequency and covered only a small frequency range. The ancestral syrinx did probably not possess any intrinsic muscles if we assume it resembled that of ostrich, emu, or cassowary [46]. Substantial frequency modulation probably only arose once tension of the vibratory tissue could be adjusted by muscular control [45].

**Fig 6:**
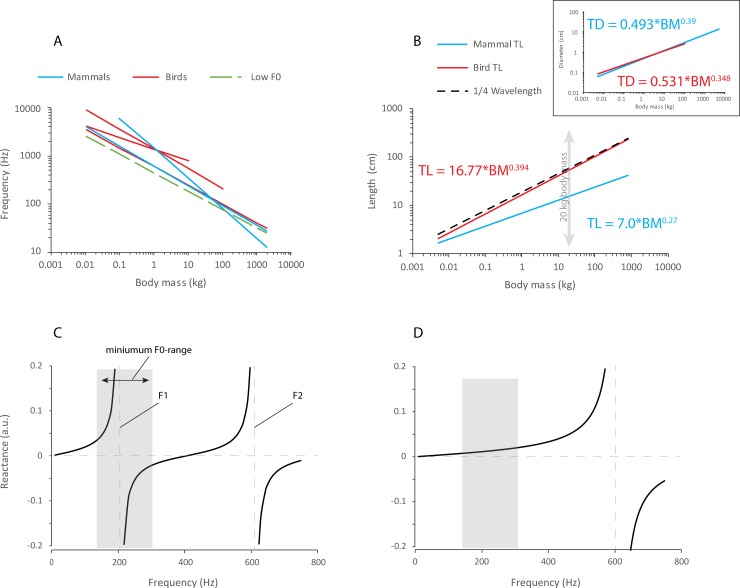
Acoustic characteristics of the avian and mammalian trachea. (A) The relationship between BM and fundamental (or dominant) frequency has been studied many times in birds and in mammals. A summary of model functions is provided in S2 Table. The regression curves for birds (red) and mammals (blue) suggest that the slope of the relationship is similar in both groups. An average regression model (F0 = 450 × BM^−0.38^) was used to estimate wavelength. Wavelength is the ratio of speed of sound (340 m/s in warm humidified air) and F0. (B) The tracheal length of birds appears to be much closer to the wavelength of F0 than that of mammals. The length and diameter of the mammalian trachea were modeled after data published by Tenney, Bartlett [17], and Moore and colleagues [76] and that of the avian trachea were modeled after data published by Hinds and Calder [18]. For a given body size, the mammalian trachea is acoustically much shorter than the avian trachea. (C, D) The graphs illustrate the relationship between vocal tract reactance and fundamental frequency for a 20-kg bird or mammal, respectively. Frequency range was established from various models listed in S2 Table. In both birds and mammals, the lowest fundamental frequency overlaps with the positive reactance range, i.e., the left side of F1. The close match between tracheal length and quarter wavelength of the fundamental frequency in birds provides the most dramatic supportive interaction. BM, body mass; F0, fundamental frequency; F1, first formant; F2, second tracheal formant.

Avian archeosaurs tend to have relatively long or very long necks. While almost no mammal has evolved more than seven cervical vertebrae, in birds, cervical vertebrae are more numerous and often elongated [74,75]. Long necks contain long tracheas. Consequently, tracheal length (i.e., suprasyringeal tracheal airspace) of birds tends to be much closer to the quarter wavelength of their voice’s lowest fundamental frequency. Whereas tracheal length is greater in birds, tracheal diameter is not different between mammals and birds (Fig 6). Most importantly, tracheal length in birds is close to the quarter wavelength of the size-predicted lowest fundamental frequency, thus enabling a boost in vocal efficiency through the overlap of positive vocal tract reactance and fundamental frequency (Fig 6C and 6D). For the first bird with a syrinx, the lowest fundamental frequency may have overlapped with the high positive reactance range just to the left of the first formant, which coincides with the most dramatic supportive interaction between source and filter. This can boost vocal efficiency.

In contrast, the vocal tract of most mammals is acoustically short, i.e., the first resonance is much higher than the lowest fundamental frequency. The wavelength of the fundamental frequency is much longer than the tracheal length (Eq 2, Fig 6D). Most living mammalian species and therapsid ancestors had short necks and neck length did not vary much [74]. Consequently, tracheal length is not sufficient for facilitating a similar boost in vocal efficiency, as was possible for long-necked birds. For example, the average adult female human trachea is about 12 cm long, and the upper vocal tract is about 14 cm long. Even if the vocal folds were located at the tracheobronchial junction (12 cm + 14 cm = 26 cm), the quarter wavelength of the fundamental frequency of a human female’s average speaking voice (210 Hz) is much longer (close to 42 cm). The discrepancy is even more pronounced for males.

With the currently available data, we present the following testable model for the evolution of the avian syrinx. The unique avian respiratory system, with its unidirectional flow through the gas exchange tissue, made gas exchange very efficient. This freed the avian bauplan from an important constraint in the neck area by allowing for more dead space (i.e., a longer trachea without a simultaneous decrease in tracheal diameter). Consequently, respiratory needs were permissive of longer necks with longer tracheas. A longer trachea shifted the avian vocal system (i.e., sound source and vocal tract) into a range for which an overlap of fundamental frequency and first tracheal resonance was possible. At this point, it became advantageous to move the sound source upstream near the tracheobronchial juncture. This model indicates that a multitude of different interacting systems must generate a permissive scenario in which novel structures for particular functions can emerge. Perhaps the evolution of a novel structure for an already existing function, such as the switch of the sound source from larynx to syrinx, particularly requires coinciding, permissive interactions [2].

## Supporting information

S1 Table First and second formants of the physical model can be estimated using the equation [(2n − 1) × c]/(4 × tube length), in which *c* is speed of sound (350 m/s) and *n* is the respective resonance frequency. The vocal fold model was coupled with 18 different tracheal lengths. The length of the tracheal tubes were chosen so that the first resonance was either lower, higher, or equal to the eigenfrequency of the physical model of 78 Hz.
(DOCX)

S2 Table Predictive value of BM for F0. BM, body mass; F0, fundamental frequency.
(DOCX)

S1 Data Excel spreadsheet containing, in separate sheets, the underlying numerical data for Figs 2A, 2B, 2C, 2D, 3A, 3B, 3C, 3D, 3E, 3F, 4A, 4B and 4C. (XLSX)

## Abbreviations

BM: body mass
F0: fundamental frequency
F1: first tracheal resonance

## References

[1] Feduccia A. The Origin and Evolution of Birds. Yale Univ. Press, 2nd edition, 1999.

[2] Kingsley EP, Eliason CM, Riede T, Li Z, Hiscock TW, Farnsworth M et al Identity and novelty in the avian syrinx. PNAS. 2018; 115: 10209–10217. 10.1073/pnas.1804586115 30249637 PMC6187200

[3] Negus VE. The comparative anatomy and physiology of the larynx. Hafner Pub. Company; 1949 New York.

[4] Bradbury JW, Vehrencamp SL. Principles of Animal Communication (2nd edition). Sunderland MA: Sinauer Associates; 2011.

[5] ten Cate C. Birdsong and Evolution in Nature’s Music In: Marler PM, Slabbekoorn H, editors. The Science of Birdsong. Elsevier; 2004 pp. 296–317.

[6] Senter P. Voices of the past: a review of Paleozoic and Mesozoic animal sounds. Hist Biol. 2008; 20: 255–287.

[7] Clarke JA, Chatterjee S, Li Z, Riede T, Agnolin F, Goller F, et al Fossil evidence of the avian vocal organ from the Mesozoic. Nature. 2016; 538: 502–505. 10.1038/nature19852 27732575

[8] Riede T, Tokuda I, Farmer CG. Subglottal pressure and fundamental frequency control in contact calls of juvenile *Alligator mississippiensis*. J Exp Biol. 2011; 214: 3082–3095. 10.1242/jeb.051110 21865521 PMC3160820

[9] Riede T, Li Z, Tokuda IT, Farmer CG. Functional morphology of the *Alligator mississippiensis* larynx and implications for vocal production. J Exp Biol. 2015; 218: 991–998. 10.1242/jeb.117101 25657203

[10] Riede T, Goller F. Peripheral mechanisms for vocal production in birds–Differences and similarities to human speech and singing. Brain Lang. 2010; 115: 69–80. 10.1016/j.bandl.2009.11.003 20153887 PMC2896990

[11] Rüppell VW. Physiologie und Akustik der Vogelstimme. J Ornith. 1933; 81: 433–542.

[12] Goller F, Larsen ON. A new mechanism of sound generation in songbirds. Proc. Natl Acad Sci. 1997; 94: 14787–14791. 9405691 10.1073/pnas.94.26.14787 PMC25115

[13] Fee MS, Shraiman B, Pesaran B, Mitra PP. The role of nonlinear dynamics of the syrinx in the vocalizations of a songbird. Nature 1998; 395: 67–71. 10.1038/25725 12071206

[14] Mindlin GB, Laje R. The Physics of Birdsong. Berlin: Springer Verlag 2005.

[15] Elemans CPH, Rasmussen JH, Herbst CT, Düring DN, Zollinger SA, Brumm H et al Universal mechanisms of sound production and control in birds and mammals. Nature communications. 2015; 6: 8978 10.1038/ncomms9978 26612008 PMC4674827

[16] Maina JN. The Biology of the Avian Respiratory System, Springer 2017.

[17] Tenney SM, Bartlett D Jr. Comparative quantitative morphology of the mammalian lung: trachea. Respiration physiology. 1967;3: 130–135. 6058337 10.1016/0034-5687(67)90002-3

[18] Hinds DS, Calder WA. Dead air space in the respiration of birds. Evol. 1971; 25: 429–440. 10.1111/j.1558-5646.1971.tb01899.x 28563117

[19] Banzett R. B., Butler J. P., Nations C. S., Barnas G. M., Lehr J. L., Jones J. H. Inspiratory aerodynamic valving in goose lungs depends on gas density and velocity. Respir Physiol. 1987; 70: 287–300. 3685652 10.1016/0034-5687(87)90011-9

[20] Brown RE, Kovacs CE, Butler JP, Wang N, Lehr J, Banzett RB. The avian lung: is there an aerodynamic expiratory valve? J. Exp. Biol. 1995; 198: 2349–2357. 9320272 10.1242/jeb.198.11.2349

[21] Schachner ER, Cieri RL, Butler JP, Farmer CG. Unidirectional pulmonary airflow patterns in the savannah monitor lizard. Nature 2014; 506: 367–370. 10.1038/nature12871 24336209

[22] Cieri RL, Craven BA, Schachner ER, Farmer CG. New insight into the evolution of the vertebrate respiratory system and the discovery of unidirectional airflow in iguana lungs. Proc Natl Acad Sci USA 2014; 111: 17218–17223. 10.1073/pnas.1405088111 25404314 PMC4260542

[23] Baer T, Lofqvist A, McGarr NS. Laryngeal vibrations: a comparison be-tween high-speed filming and glottographic techniques. J Acoust Soc Am. 1983; 73: 1304–1308. 6853841 10.1121/1.389279

[24] Matsushita H. The vibratory mode of the vocal folds in the excised larynx. Folia Phoniatr. (Basel). 1975; 27: 7–18. 1183913 10.1159/000263963

[25] Alipour F, Scherer RC, Finnegan E. Pressure-flow relationships during phonation as a function of adduction. J Voice 1997; 11: 187–194. 9181542 10.1016/s0892-1997(97)80077-x

[26] Zhang Z, Neubauer J, Berry DA. The influence of subglottal acoustics on laboratory models of phonation. J Acoust Soc Am. 2006; 120: 1558–1569. 17004478 10.1121/1.2225682

[27] Brown C, Alipour F, Berry DA, Montequin D. Laryngeal biomechanics and vocal communication in the squirrel monkey (*Saimiri boliviensis*). J Acoust Soc Am. 2003; 113: 2114–2126. 12703722 10.1121/1.1528930

[28] Titze IR. Principles of Voice Production. Author, 2nd Edition, National Center for Voice and Speech 2000.

[29] Frey R, Riede T. The anatomy of vocal divergence in North American elk and European red deer. J Morph. 2013; 274: 307–319. 10.1002/jmor.20092 23225193 PMC3928815

[30] Pasch B, Tokuda I, Riede T. Grasshopper mice employ distinct vocal production mechanisms in different social contexts. Proc Roy Soc Lond B. 2017; 284: 20171158. 10.1098/rspb.2017.1158 PMC5543235 28724740

[31] Riede T, Beckers GJL, Blevins W, Suthers RA. Inflation of the Esophagus and Vocal Tract Filtering in Ring Doves. J Experimental Biology 2004; 207: 4025–4036. 10.1242/jeb.01256 15498948

[32] Riede T, Suthers RA, Fletcher N, Blevins W 2006 Songbirds tune their vocal tract to the fundamental frequency of their song. Proc Nat Acad Sci, 103: 5543–5548. 10.1073/pnas.0601262103 16567614 PMC1459391

[33] Riede T, Eliason CM, Miller EH, Goller F, Clarke JA. Coos, booms, and hoots: The evolution of closed‐mouth vocal behavior in birds. Evolution. 2016; 70: 1734–1746. 10.1111/evo.12988 27345722

[34] Fletcher N, Riede T, Suthers RA. Model for vocalization by a bird with distensible vocal cavity and open beak. J Acoust Soc Am. 2006; 119: 1005–1011. 16521762 10.1121/1.2159434

[35] Titze IR. The physics of small-amplitude oscillation of the vocal folds. J Acoust Soc Am. 1988; 83: 1536–1552. 3372869 10.1121/1.395910

[36] Fletcher N. Autonomous vibration of simple pressure controlled valves in gas flows. J. Acoust. Soc. Am. 1993; 93: 2172–2180.

[37] Titze IR. Nonlinear source–filter coupling in phonation: Theory. J Acoust Soc Am. 2008; 123: 2733–2749. 10.1121/1.2832337 18529191 PMC2811547

[38] Titze IR, Riede T, Popollo P. Vocal exercises to determine nonlinear source–filter interaction. J Acoust Soc Am. 2008; 123: 1902–1915. 10.1121/1.2832339 18396999 PMC2677316

[39] Riede T, Tokuda I, Munger JB, Thomson SL. Mammalian laryngeal air sacs add variability to the vocal tract impedance: Physical and computational modeling. J Acoust Soc Am. 2008; 124: 634–647. 10.1121/1.2924125 18647005 PMC2677336

[40] Riede T, Goller F. Functional morphology of the sound generating labia in the syrinx of two songbird species. J. Anat. 2010; 216: 23–36. 10.1111/j.1469-7580.2009.01161.x 19900184 PMC2807973

[41] Riede T, Goller F. Morphological basis for the evolution of acoustic diversity in oscine songbirds. Proc Roy Soc B. 2014; 281: 20132306. 10.1098/rspb.2013.2306 PMC3924064 24500163

[42] Thomson SL, Mongeau L, Frankel SH. Aerodynamic transfer of energy to the vocal folds. J Acoust Soc Am 2005; 118, 1689–1700. 16240827 10.1121/1.2000787

[43] Zhang Z, Neubauer J, Berry DA. Aerodynamic and acoustically driven modes of vibration in a physical model of the vocal folds. J Acoust Soc Am. 2006; 120: 2841–2849. 17139742 10.1121/1.2354025

[44] Neubauer J, Zhang Z, Miraghaie R, Berry DA. Coherent structures of the near field flow in a self-oscillating physical model of the vocal folds. J Acoust Soc Am. 2007; 121: 1102–1118. 17348532 10.1121/1.2409488

[45] Goller F, Riede T. Integrative physiology of fundamental frequency control in birds. J Physiol. (Paris) 2013; 107: 230–242. 23238240 10.1016/j.jphysparis.2012.11.001 PMC3674193

[46] King AS. Functional anatomy of the syrinx In: King AS, McLelland J, editors. Form and Function in Birds. Vol. 4 London, UK: Academic Press 1989; pp. 105–192.

[47] Ishizaka K, Flanagan JL. Synthesis of voiced sounds from a two-mass model of the vocal cords. Bell Lab Tech J. 1972; 51: 1233–1268.

[48] Titze IR, Palaparthi A. Sensitivity of source-filter interaction to specific vocal tract shapes. IEEE Trans Audio Speech Signal Process. 2016; 24: 2507–2515. 10.1109/taslp.2016.2616543 PMC9390861 35990794

[49] Titze IR, Palaparthi AKR, Smith SL. Benchmarks for time-domain simulation of sound propagation in soft-walled airways: steady configurations. J Acoust Soc Am. 2014; 136: 3249–3261. 10.1121/1.4900563 25480071 PMC4257962

[50] Myers J.A. Studies on the syrinx of Gallus domesticus. J Morphol. 1917; 29: 165–214.

[51] Miller AH. Vocal apparatus of North American Owls. Condor. 1934; 36: 204–213.

[52] Paulsen K. Das Prinzip der Stimmbildung in der Wirbelthierreihe und beim Menschen. Akademische Verlagsgesellschaft, Frankfurt am Main, 1967.

[53] Garcia SM, Kopuchian C, Mindlin GB, Fuxjager MJ, Tubaro PL, Goller F. Evolution of vocal diversity through morphological adaptation without vocal learning or complex neural control. Curr Biol. 2017; 27: 2677–2683. 10.1016/j.cub.2017.07.059 28867206 PMC5599221

[54] Elemans CPH, Laje R, Mindlin GB, Goller F. Smooth operator: avoidance of subharmonic bifurcations through mechanical mechanisms simplifies song motor control in adult zebra finches. J Neurosci. 2010; 30: 13246–13253. 10.1523/JNEUROSCI.1130-10.2010 20926650 PMC3487382

[55] Ballentijn MR, tenCate C. Sound production in the collard dove: a test of the ‘whistle’ hypothesis. J Exp Biol. 1998; 201: 1637–1649. 9556544 10.1242/jeb.201.10.1637

[56] Arneodo M, Mindlin GB. Source-tract coupling in birdsong production. Phys Rev E. 2009; 79: 061921. 10.1103/PhysRevE.79.061921 19658538

[57] Van den Berg J. Sound production in isolated human larynges. Ann NY Acad Sci. 1968; 155: 18–27. 5258045 10.1111/j.1749-6632.1968.tb56745.x

[58] Austin M, Titze IR. The effect of subglottal resonance upon vocal fold vibration. J Voice 1997; 12: 391–402. 10.1016/s0892-1997(97)80034-3 9422272

[59] Herzel H, Berry D, Titze IR, Steinecke IR. Nonlinear dynamics of the voice: Signal analysis and biomechanical modeling. Chaos 1995; 5: 30–34. 10.1063/1.166078 12780151

[60] Rothenberg M. Acoustic interaction between the glottal source and the vocal tract In Vocal Fold Physiology, edited by Stevens K. N. and Hirano M. University of Tokyo Press, Tokyo 1981, pp. 305–328.

[61] Dürrwang, R. Funktionelle Biologie, Anatomie und Physiologie der Vogelstimme. PhD dissertation, University of. Basel, Switzerland. 1974.

[62] Abs M. Zur Bioakustik des Stimmbruchs bei Vögeln. Zool Jb Physiol. 1980; 84: 289–382.

[63] Brittan-Powell EF, Dooling RJ, Larsen ON, Heaton JT. Mechanisms of vocal production in budgerigars (*Melopsittacus undulatus*). J Acoust Soc Am. 1997; 101: 578–589. 9000746 10.1121/1.418121

[64] Elemans CPH, Muller M, Larson ON, Leeuwen JL 2009 Amplitude and frequency modulation of sound production in a mechanical model of the avian syrinx. J Exp Biol 212, 1212–1224. 10.1242/jeb.026872 19329754

[65] Gross WB. Voice production by the chicken. Poultry Sci. 1964; 43: 1005–1008.

[66] Nowicki S. Vocal tract resonances in oscine bird sound production: Evidence from birdsongs in a helium atmosphere. Nature 1987; 325: 53–55. 10.1038/325053a0 3796738

[67] Maxfield L, Palaparthi A, Titze IR. New evidence that nonlinear source-filter coupling affects harmonic intensity and F0 stability during instances of harmonics crossing formants. J Voice 2017; 31, 149–156. 10.1016/j.jvoice.2016.04.010 27501922 PMC5292312

[68] Palaparthi A, Maxfield L, Titze IR. Estimation of source-filter interaction regions based on electroglottography. J Voice. 2017; 10.1016/j.jvoice.2017.11.012 PMC6014870 29277351

[69] Mergell P, Herzel H. Modelling biphonation—The role of the vocal tract. Speech Commun. 1997; 22: 141–154.

[70] Forstmeier W, Kempenaers B, Meyer A, Leisler BA. Novel song parameter correlates with extra-pair paternity and reflects male longevity. Proc Roy Soc B. 2002; 269: 1479–1485. 10.1098/rspb.2002.2039 PMC1691048 12137578

[71] Ritschard M, Riebel K, Brumm H. Female zebra finches prefer high-amplitude song. Anim Behav. 2010; 79: 877–883.

[72] Lee MSY, Cau A, Naish D, Dyke GJ. Sustained miniaturization and anatomical innovation in the dinosaurian ancestors of bird. Science 2014; 345: 562 10.1126/science.1252243 25082702

[73] Daley M, Goller F. Tracheal length changes during zebra finch song and their possible role in upper vocal tract filtering. J. Neurobiol. 2004; 59: 319–330. 10.1002/neu.10332 15146548

[74] Müller J, Scheyer TM, Head JJ, Barrett PM, Werneburg I, Ericson PGP, Pol D, Sánchez-Villag MR. Homeotic effects, somitogenesis and the evolution of vertebral numbers in recent and fossil amniotes. PNAS 2009; 107: 2118–2123 10.1073/pnas.0912622107 PMC2836685 20080660

[75] Taylor MP, Wedel MJ. Why sauropods had long necks; and why giraffes have short necks. PeerJ 2013; 1: e36 10.7717/peerj.36 23638372 PMC3628838

[76] Moore C, Moore M, Trumble S, Niemeyer M, Lentell B, McLellan W et al A comparative analysis of marine mammal tracheas. Journal of Experimental Biology. 2013; 217, 1154–1166. 10.1242/jeb.093146 24311807

