# Supplementary material for: The evolution of the syrinx: An acoustic theory
Source: PLoS Biol. 2019 Feb 7;17(2):e2006507. doi: 10.1371/journal.pbio.2006507 (PMC6366696; doi:10.1371/journal.pbio.2006507)
Supplement: S1 Table — The vocal fold model was coupled with 18 different tracheal lengths. The length of the tracheal tubes were chosen so that the first resonance was either lower, higher, or equal to the eigenfrequency of the physical model of 78 Hz. (DOCX) [file pbio.2006507.s001.docx]

**S1 Supplemental Table**

| Tracheal length (cm) | First resonance, F1 (Hz) | Second resonance, F2 (Hz) |
| --- | --- | --- |
| 5.1 | 1708 | 5126 |
| 10.2 | 854 | 2563 |
| 15 | 583 | 1750 |
| 20 | 437 | 1312 |
| 41 | 213 | 640 |
| 60 | 145 | 437 |
| 80 | 109 | 328 |
| 100 | 87 | 262 |
| 119 | 73 | 220 |
| 142 | 61 | 184 |
| 159 | 55 | 165 |
| 180 | 48 | 145 |
| 200 | 43 | 131 |
| 220 | 40 | 119 |
| 256 | 34 | 102 |
