# Supplementary material for: The evolution of the syrinx: An acoustic theory
Source: PLoS Biol. 2019 Feb 7;17(2):e2006507. doi: 10.1371/journal.pbio.2006507 (PMC6366696; doi:10.1371/journal.pbio.2006507)
Supplement: S2 Table — BM, body mass; F0, fundamental frequency. (DOCX) [file pbio.2006507.s002.docx]

**S2 Supplemental Table**

| **Source** | **Birds** | **Mammals** |
| --- | --- | --- |
| Wallschlaeger 1980 | F0 = 7204 * BM^-0.24^ |  |
| Goller, Riede 2013 | Minimum F0= 600 * BM^-0.39^  Maximum F0 = 9800 * BM^-0.35^ |  |
| Fletcher 2004 | F0 = 1404 * BM^-0.41^ | F0 = 1404 * BM^-0.41^ |
| Tembrock 1996 |  | F0 = 1443 * BM^-0.623^ |
| Martin et al. 2016 |  | Terrestrial mammals  Minimum F0: 617 * BM^-0.41^  Terrestrial mammals  Maximum F0: 5.3 * BM^-0.38^ |
